# Uncommon Presentation of Clostridioides difficile in the Small Bowel: A Case Report and Review of Literature

**DOI:** 10.7759/cureus.43460

**Published:** 2023-08-14

**Authors:** Sunanda Tah, Saqib Khan, Sarang Kashyap

**Affiliations:** 1 Surgery, Saint James School of Medicine, Arnos Vale, VCT; 2 Surgery, Beckley Appalachian Regional Healthcare (ARH) Hospital, Beckley, USA; 3 Surgery, Avalon University School of Medicine, Willemstad, CUW

**Keywords:** diarrhea, loop ileostomy, pittsburgh protocol, small bowel, community-acquired clostridium difficile infection (cdi), clostridium difficile infection treatment

## Abstract

*Clostridioides difficile* infection (CDI) is a prevalent source of hospital-acquired diarrhea. The most common presentation of CDI is colitis. In cases of fulminant colitis/toxic megacolon, a colectomy and end ileostomy are part of the treatment plan. There is evidence to suggest that it may be beneficial to surgically treat severe complex CDI by constructing a loop ileostomy for fecal stream diversion followed by colonic lavage, also referred to as the Pittsburgh protocol, which has demonstrated decreased death rates in this patient population. In our case study, we present a rare case of a 60-year-old female patient diagnosed with fulminant small bowel CDI requiring resection of the necrotic small bowel. This was followed by creating an ileostomy and the Pittsburgh protocol, leading to a complete recovery. With an increasing incidence of CDI, it is important to be aware of the small bowel *C. difficile* infection and its treatment.

## Introduction

*Clostridioides difficile* continues to be the most common cause of nosocomial diarrhea [1]. *Clostridioides difficile *infection (CDI) generally occurs in individuals who have recently received antibiotics that alter the flora of the colon, allowing the *C. difficile* bacteria to flourish and produce toxins. The two main toxins produced by *C. difficile* are toxins A and B. These toxins damage the lining of the colon, causing inflammation and diarrhea. Individuals who are highly susceptible to CDI tend to be of advanced age, on antacids, or have inflammatory bowel disease [2]. However, community-acquired CDI is also known to occur in healthy individuals. *Clostridioides difficile* fulminant colitis is a severe infection characterized by the presence of hypotension, shock, or toxic megacolon. Fulminant *C. difficile* colitis is uncommon, with an incidence of 3% to 8% [3]. It is characterized by a mortality rate of 34% to 80% with surgical management vs. a 50% to 70% mortality rate in non-surgical care [4]. Standard treatment of fulminant *C. difficile *colitis includes aggressive antibiotic therapy with vancomycin and metronidazole, along with colectomy and ileostomy. In addition to the standard medical treatment for fulminant CDI, another procedure popularly known as the 'Pittsburgh protocol' focuses on colonic lavage after the creation of a loop ileostomy. The reported mortality in this set of patients was 19% [5].

We present a rare case of severe small bowel CDI in a patient without any history of inflammatory bowel disease (IBD) or prior gastrointestinal (GI) surgeries. The patient was treated with small bowel resection, the creation of an ileostomy, antibiotic therapy, and the Pittsburgh protocol. This case illustrates that even though it may be rare, CDI can infect the small bowel and progress to the fulminant stage. This presents a challenge in the diagnosis as well as the surgical and medical management of these patients.

## Case presentation

A 60-year-old female presented to the emergency department with non-bloody diarrhea, vomiting, poor appetite, abdominal cramps, and generalized weakness for four days. Past history was significant for IV drug abuse, neuropathy, deep vein thrombosis, anxiety, and depression. A month prior to the present admission, the patient was treated for pneumonia. 

**Figure 1:**
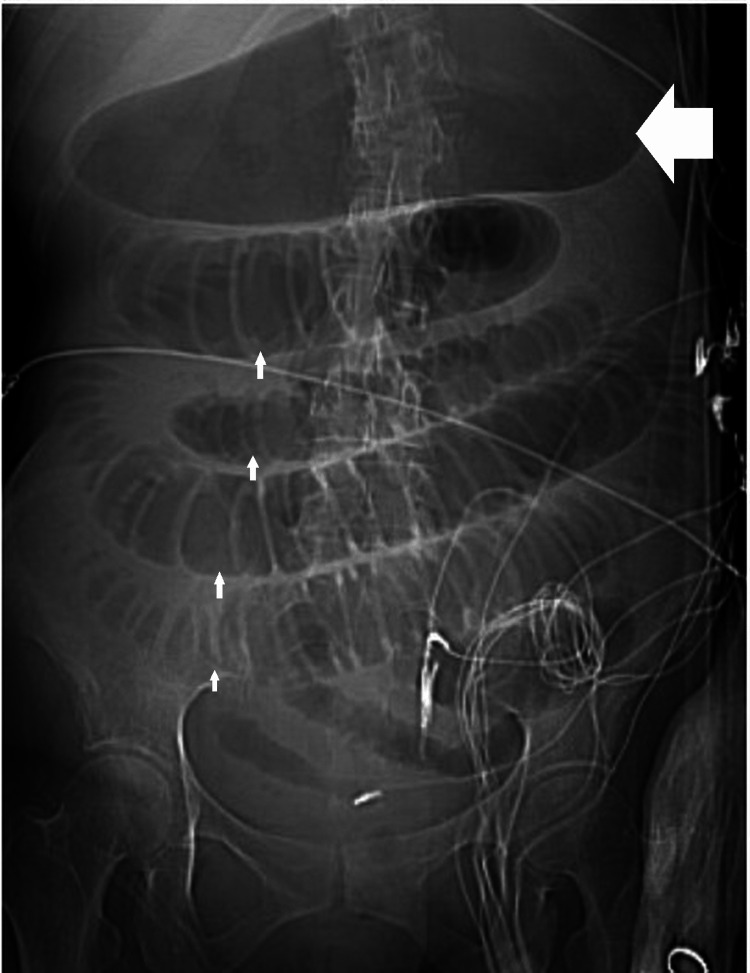
Scout film from the abdomen/pelvis CT revealed distention of the stomach (horizontal arrow) and distended small bowel loops (vertical arrows) consistent with small bowel obstruction

**Figure 2:**
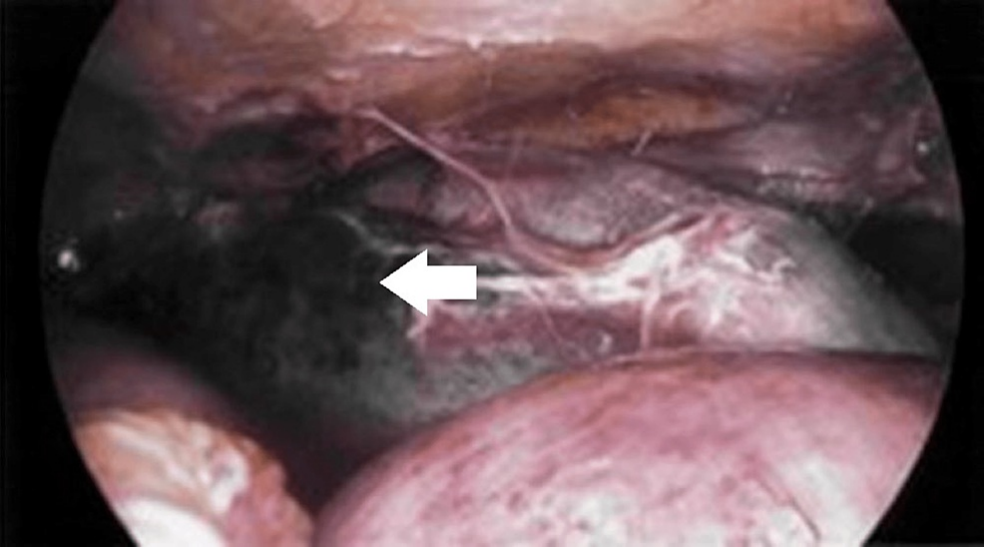
Intraoperative image taken during diagnostic laparoscopy showing necrotic small bowel (white arrow)

At the time of presentation, the patient was stable. There were no signs of peritonitis. However, she deteriorated clinically over the next two to three days. Hypotension and tachycardia ensued. Leukocytosis and lactic acidosis worsened. The stool assay was positive for *C. difficile*. She was started on standard medical management with oral vancomycin and IV metronidazole. The CT scan revealed a small bowel ileus, but none of the colon was distended (Figure 1). Despite maximal medical management, her clinical condition did not improve. She underwent a diagnostic laparoscopy that revealed a necrotic small bowel (Figure 2) but a healthy-appearing colon. An exploratory laparotomy and resection of the small bowel were performed. The length of the small bowel resected was 70 cm. The length of the terminal ileum towards the ileocecal valve that was left behind was approximately 20.32 cm. The patient subsequently underwent a modified loop ileostomy. Catheters were placed proximally and distally in the bowel loops. One catheter was placed in the distal loop to provide polyethylene glycol and vancomycin to the distal bowel and colon. Another catheter was placed in the proximal small bowel to administer vancomycin to the proximal small bowel. The pathology report confirmed pseudomembranes in the resected small bowel. *Clostridioides difficile* was confirmed from ileostomy samples.

The patient had ileus for a prolonged period, which made the administration of oral vancomycin difficult. In addition to the IV metronidazole treatment, vancomycin was administered through the long rubber catheter. Polyethylene glycol and vancomycin were flushed through the distal loop of the ileostomy daily until the WBC count normalized. The patient had a fecal management system in place to reduce source contamination. The patient's total hospital stay was about two months. She recovered well and was eventually transferred to a nursing home.

## Discussion

It is important to note that *C. difficile* can infect more than just the colon. There have been 81 cases of small bowel CDI in the literature [1]. Small bowel CDI presents a unique challenge in many ways. Patients have ileus, making it difficult to institute oral vancomycin, which is the standard of care. Another challenge is the high mortality rate of approximately 30% [6]. 

Patients with this unusual presentation typically have some type of predisposition, with colectomy being the greatest risk factor since it alters the small bowel flora by disturbing normal peristalsis and the function of the ileocecal valve [7]. Post-colectomy, the microbiome of the small intestine becomes similar to that of the colon, making it prone to a *C. difficile* infection, especially after antibiotic use [8]. Colonization of the small bowel can also occur because the mechanical action of the ileocecal valve is reduced or lost after surgery [9]. In our case, however, the patient had no prior colonic surgeries, making this case even more unique. We hypothesize that *C. difficile* retro-migrated from the colon to the small bowel across the ileocecal valve.

The standard of care for fulminant CDI colitis includes high doses of oral vancomycin and IV metronidazole. In patients with paralytic ileus, vancomycin enemas can be beneficial since oral vancomycin may not reach the colon. Surgical management, which includes total colectomy with end ileostomy, is indicated for critically ill patients unresponsive to the antibiotic regimen. Despite standard medical care, including total colectomy with end ileostomy, mortality remains high [10].

As demonstrated in our case, early diagnostic laparoscopy and/or laparotomy should be encouraged in patients who do not respond to medical management or clinically worsen despite treatment. For our patient, we used a joint approach in which the necrotic small bowel was resected, followed by the Pittsburgh protocol which consists of colonic lavage with warm polyethylene glycol through the loop ileostomy, followed by vancomycin flushes. Oral vancomycin was also continued for enteritis after the ileus resolved. Although the colon appeared normal during surgery, CDI of the colon affects the mucosal surface and should be treated until the patient improves clinically.

## Conclusions

While small intestinal *C. difficile* remains uncommon, its occurrence could rise due to increasing *C. difficile* infections globally. Patients with small bowel CDI exhibit symptoms similar to those of *C. difficile* colitis. Colon appearance might be unaffected on imaging. If patients worsen or don't improve with medical treatment, diagnostic laparoscopy or laparotomy should be considered. Treatment lacks established guidelines due to its rarity, but our case suggests success by combining the Pittsburgh protocol, necrotic small bowel resection, and ongoing antibiotics. This approach could be considered for such cases.
